# Biochemical and Molecular Aspects of Vascular Adrenergic Regulation of Blood Pressure in the Elderly

**DOI:** 10.1155/2012/915057

**Published:** 2011-09-22

**Authors:** William E. Schutzer, Scott L. Mader

**Affiliations:** Portland VA Medical Center and Research Service—RD 26, Oregon Health and Science University School of Medicine, P.O. Box 1034, Portland, OR 97201, USA

## Abstract

Hypertension, orthostatic hypotension, arterial insufficiency, and atherosclerosis are common disorders in the elderly that lead to significant morbidity and mortality. One common factor to these conditions is an age-related decline in vascular beta-adrenergic receptor-mediated function and subsequent cAMP generation. Presently, there is no single cellular factor that can explain this age-related decline, and thus, the primary cause of this homeostatic imbalance is yet to be identified. However, the etiology is clearly associated with an age-related change in the ability of beta-adrenergic receptor to respond to agonist at the cellular level in the vasculature. This paper will review what is presently understood regarding the molecular and biochemical basis of age-impaired beta-adrenergic receptor-mediated signaling. A fundamental understanding of why **β**-AR-mediated vasorelaxation is impaired with age will provide new insights and innovative strategies for the management of multiple clinical disorders.

## 1. Introduction

### 1.1. Clinical Relevance

Life expectancy has increased during the past century, and this has led to a dramatic increase in the aging population. The number of Americans over 65 is expected to double from the years 2000 to 2030: in 2000, there were 34.8 million over the age of 65 (12% of the population), and in 2030, it is predicted that there will be 70.3 million in this age group (representing 20% of the population) [1]. This change in population dynamics presents substantial medical issues, as aging is a primary independent risk factor for development of cardiovascular disorders. The fact that aging contributes to cardiovascular morbidity is not novel. Sir William Osler (1849–1919), one of the founders of Johns Hopkins University Hospital, stated in his textbook that, “Longevity is a vascular question… a man is only as old as his arteries.” From a strictly aging/vascular perspective, numerous recent articles have described changes in anatomical and histological properties with age (e.g., see [2]) as well as biophysical alterations, such as with changes in pulse wave velocity with age (as described in [3]). However, one possibility to consider is that these aforementioned changes are likely due to aging-mediated alterations in the molecular and biochemical factors that determine vascular tone. 

Vascular tone is regulated by both the intimal (endothelial) and medial (vascular smooth muscle) layers as well as through interlayer interactions. Age-related changes in the structure and function of each layer is well documented [4, 5]. Smooth muscle cells represent the major arterial cell population, and these cells highly express adrenergic receptors that mediate smooth muscle tone. Thus, adrenergic receptors are important regulators of cardiovascular physiology. Although all three beta adrenergic receptor (*β*-AR) subtypes, *β*1, *β*2, and *β*3, are found in vascular smooth muscle cells, the *β*2-AR subtype is by far the most highly expressed [6]. Of specific relevance to this paper is that the vascular *β2*-AR exhibits an age-related decline in signaling with advancing age that leads to impaired vasorelaxation. In contrast, the intrinsic ability for vascular muscle contraction is generally maintained throughout the aging process [7]. This change is important, because it may allow for multiple age-associated clinical conditions such as hypertension, arterial insufficiency, orthostatic hypotension, and arteriosclerosis. The underlying change is hypothesized to be a decrease in *β*-AR-stimulated cAMP production. Therefore, conditions associated with altered cAMP production are likely affected. To this end, the *β*-AR is a target for many medications prescribed to the elderly [8] and are used to manage hypertension, angina, postmyocardial infarction risk, congestive heart failure, glaucoma, tremor, arrhythmias, and chronic obstructive pulmonary disorders [9].

### 1.2. Basic Science Relevance

Beta-AR-mediated signal transduction pathways are well described, but new discoveries continue to impart complexity (see [10] and following sections). At present, no change in any one factor in the *β*-AR signal cascade has been identified to fully explain the impaired *β*-AR vascular function observed with aging. Instead, the cause of the change is likely multifactorial. This notion is supported due to intricate nature of *β*-AR signaling and two biochemical findings: first is that expression of *β*2-AR does not change with age [11], and secondly, although drugs that activate *β*-ARs do *not* elicit complete vasorelaxation with advancing age, drugs that act on proteins postreceptor in the signaling cascade *do* [12]. That is, the physiologic factors that mediate vasorelaxation cannot completely dilate blood vessels with advancing age; however, the molecular and cellular/anatomic machinery—postreceptor—remains fully functional.

## 2. Contraction/Relaxation Vascular Pharmacodynamics

### 2.1. Mechanisms of Vascular Contraction/Relaxation

Vascular tone is physically established in the medial layer of blood vessels, which is almost entirely composed of vascular smooth muscle cells. Numerous agents (epinephrine, norepinephrine, acetylcholine, angiotensin II, nitric oxide, etc.) function through their cognate receptors localized at vascular smooth muscle, and/or endothelial cells and influence an elaborate network of signal transduction pathways that yields homeostatic control [13]. The molecular mechanisms regulating smooth muscle contraction and relaxation are beyond the scope of this paper; however, excellent reviews are found elsewhere [14, 15].

### 2.2. Vascular *β*-AR Signaling

In blood vessels, the *β*-AR signal transduction cascade mediates smooth muscle vasorelaxation. Activation of the *β*-AR stimulates the dissociation of the G protein, G*α*s, from the *βγ* subunit. The G protein *βγ* subunit can also affect various membrane and/or organelle channels whose action can rapidly alter the ionic milieu of the cell. After uncoupling from the *β*-AR, G*α*s becomes activated by exchanging GDP for GTP. The activated form of G*α*s triggers adenylyl cyclase to convert ATP into cAMP [16]. Two molecules of this second messenger bind one regulatory subunit of protein kinase A (PKA). Structurally, PKA is a tetrameric kinase made up of regulatory and catalytic subunit dimers. Functionally, PKA is a multipurpose kinase that controls numerous cellular events by phosphorylating protein targets. PKA is distributed to multiple discrete intracellular compartments via the function of A-kinase anchoring proteins (AKAP) [17]. *β*-AR activation also initiates G protein receptor kinase (GRK) function. GRKs are a family of kinases that phosphorylate, *β*-ARs [18]. Phosphorylated *β*-ARs are targets for still another group of proteins, the *β*-arrestins that desensitize *β*-ARs and mediate internalization, which leads to receptor recycling and/or degradation. In addition, *β*-arrestins can serve as scaffolds and adaptors for other kinases such as extracellular signal-regulated kinase (ERK), Src, and Raf that regulate several cellular pathways that result in the activation of MAP kinases [19].

Identifying a possible locus for the age-related decline in *β*-AR function has proven elusive due to the complexity of this cascade. Three subtypes of the *β*-AR (*β*1-AR, *β*2-AR, and *β*3-AR) are found in vascular smooth muscle cells [20]. Also, at least nine different isoforms of adenylyl cyclase have been identified [21, 22], along with at least six GRK [23], three arrestin [24], and multiple types of AKAP [25] isoforms are known to exist. Similarly, multiple G*α*s-modifying proteins are also known to alter *β*-AR signaling [26]. Therefore, to understand the mechanism(s) of impaired *β*-AR function with age, recent research has focused on proteins that interact with the *β*-AR, directly or indirectly, that may be critical for optimal receptor signaling. Investigations on age-related changes in various modifications to the *β*-AR, such as phosphorylation-mediated desensitization, scaffolding proteins that form postreceptor signalosomes, or proteins that directly interact and affect *β*-AR function may provide insight to explain the change.

## 3. Age-Related Changes in Vascular Function

The initial observation of an aging effect on vasorelaxation was made in the early 1970s involving analysis of vascular smooth muscle pharmacodynamics in general. Blood vessels from 6-month-old animals relaxed 90% less to isoproterenol as compared to blood vessels from 1-month-old animals [27, 28]. To explain this physiologic change, biochemical analysis found that both basal- and isoproterenol-mediated cAMP synthesis significantly declined in isolated aorta with advancing age. This was in contrast to adenylyl cyclase and phosphodiesterase activity that was essentially unaffected by age. From these results, it was concluded that the decreased ability of isoproterenol to elevate intracellular cAMP concentration, and thus relax aortas from older rats was predicated by an “upstream” change in the *β*-AR itself, rather than a “downstream” change specific to adenylyl cyclase [29]. From these initial observations, numerous researchers have evaluated the aging vasculature in an attempt to uncover the mechanism of this change in *β*-AR function with age. As described above, the *β*-AR signaling cascade is multifaceted, including numerous protein factors, each of which exists with multiple subtypes. The following is a discussion of what is currently known regarding the mechanism of the age-related change in *β*-AR function, organized in a sequential manner, starting upstream with the *β*-AR itself and continuing through the cascade as we currently understand it.

### 3.1. Beta-Adrenergic Receptors

Presently, three distinct subtypes of the *β*-AR have been identified in mammals, (*β*1-AR, *β*2-AR, and *β*3-AR [20]: crystalline structures (including affect of agonist occupancy) have recently been described [30]. Most of the literature demonstrates that the primary *β*-AR subtype in the vasculature is the *β*2-AR [31], but both *β*1-AR and *β*3-AR are present and mediate vasorelaxation [32]. The overall distribution, and relative proportion, of each *β*-AR subtype varies across vascular beds [33, 34]. These three *β*-AR subtypes work in concert to alter vascular tone in a complementary manner, as they all couple to G*α*s and promote cAMP production [35].

The vascular adrenergic receptor subtypes behave differently after exposure to agonist. Both *β*1-AR and *β*2-AR desensitize with activation due to the function of GRKs, PKA, as well as other kinases and/or factors [36, 37]. Interestingly, agonist exposure to the *β*2-AR also initiates the transformation of its G protein-coupling selectivity from G*α*s to G*α*i [38]. This phenomenon has been documented in the heart [39] and multiple cultured cell lines [40]. When the *β*2-AR is linked to G*α*i, one cellular event that occurs is an inhibition of adenylyl cyclase activity that is manifested as a reduction of intracellular cAMP concentration [41]. There are no data to suggest that *β*1-AR changes its G*α* protein subtype coupling preference. In contrast, the *β*3-AR does not appear to undergo desensitization, as it lacks regulatory phosphorylation sites for GRKs, PKA, or other kinases [42]. This characteristic may allow for prolonged signaling compared to *β*1-AR- and *β*2-AR-mediated effects. Although expression levels of the three adrenergic receptor types is differential, with *β*2 being the most predominant subtype, and *β*3 being the least expressed, patterns of function could be altered with pathology. For instance, under maintained stimulation, *β*1-AR is predicted to desensitize, *β*2-AR is predicted to desensitize and further inhibit cAMP production through its linkage to G*α*i [43], and *β*3-AR is predicted to possibly represent a functional alternative for cAMP production [6].

We have evaluated whether an agonist-mediated change in *β*2-AR/G protein coupling observed in the heart could explain the age-related change in vasorelaxation [12]. Using pertussis toxin (pertussis toxin irreversibly ADP ribosylates and inactivates G*α*i) to block the coupling of activated *β*2-AR to G*α*i in aortae isolated from Fischer 344 rats of increasing age, it was found that this treatment did not alter the age-related decline in relaxation. However, a population of *β*2-AR coupled to G*α*i was found, as pertussis toxin treatment improved *β*2-AR-mediated vasorelaxation in aortae for all ages in equal proportion. Changes in vascular *β*3-AR function with aging are unknown. However, left ventricular function and age-related heart failure were highly correlated with *β*3-AR expression in rats [44]. These findings suggest that further investigation is warranted to characterize AR subtype expression patterns and *β*3-AR function throughout the vasculature.

Because the *β*2-AR is the most highly expressed subtype in the vasculature, much interest is the finding that *β*2-AR sensitivity substantially declines with age [45]. Results found that in aortic preparations from 1-month-old animals, 64% of the *β*-ARs were in the high affinity state. This compared to 40%, and 0% high affinity *β*-ARs for 6- and 24-month-old animals, respectively. To explain these results, age-related changes in the content of *β*2-AR bound to G*α*s was examined: *β*2 AR : G*α*s complexes were found *only* in aortic preparations from 1-month-old animals. These data strongly suggest that there is a substantial decline in high-affinity *β*2-AR with advancing age. This change did not appear to be related to a decline in the presence of *β*-ARs at the membrane (further confirmed [11]) or caused by a switch in G protein coupling as occurs in cardiac tissue [12].

**Figure 1:**
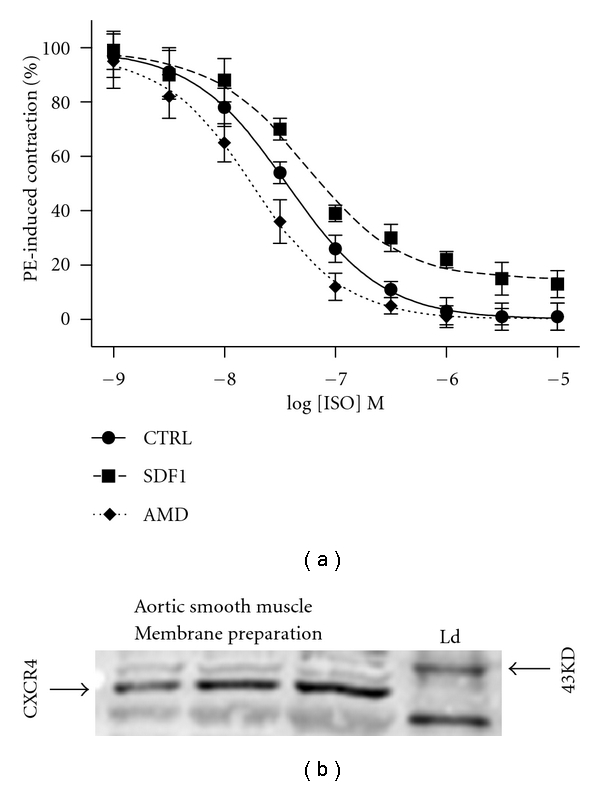
Vascular smooth muscle and chemokine receptor subtype 4 (CXCR4). (a) Aortae from 2-month-old animals (*n* = 5) were isolated and mounted on an apparatus to measure vascular reactivity as described [12]. Vessels were exposed to three treatments. The control (CTRL) treatment was that vessels were contracted with phenylephrine (PE) and allowed to stabilize (approximately 10 minutes) that was followed with relaxation stimulated by increasing doses of isoproterenol (ISO). The stromal cell derived factor (SDF1; ligand for the CXCR4 receptor also known as CXCL12) treatment was similar except that after the PE-mediated contraction stabilized, 100 ng/mL SDF1 was added. Following a 5-minute SDF1 incubation, relaxation was stimulated by increasing doses of ISO. SDF1 did not alter the tone produced by PE alone. The AMD-3100 (AMD; a CXCR4 specific antagonist) treatment occurred with a 45 minute, 10 *μ*M AMD incubation preceding the PE-mediated contraction and stabilization (10 minutes), which was followed by relaxation stimulated by increasing doses of ISO. AMD treatment did not effect PE-mediated contraction as compared to CTRL. Also conducted, but not shown, is that addition of AMD blocked the effect of SDF1 thereby implicating a direct effect of CXCR4 on impairing *β*-AR-mediated vasorelaxation. Data are expressed as percent of PE-induced contraction. The doses for AMD, and SDF1 were used as per LaRocca et al. [50]. (b) Aortic smooth muscle medial layers were homogenized, and membrane-specific fractions were prepared for western blotting as described [51]. Increasing concentrations (5 *μ*g, 10 *μ*g, and 20 *μ*g; as determined with BCA analysis) of total protein extracts were loaded in each lane. A specific antibody for CXCR4 (Abcam; Cambridge, Mass, USA) was used to visualize expression of CXCR4 that is predicted to locate at approximately 39 kilodaltons (kD). Shown also is a size-indicating ladder (Ld).

Another interesting possibility to explain the age-related decline in *β*2-AR signaling is the possibility that *β*-AR can form hetero- and homodimers, and this could alter signaling fidelity. Mercier et al. [46] showed that *β*-ARs likely exist as either *β*1-AR :  *β*1-AR homodimers, or *β*1-AR : *β*2-AR heterodimers, and they suggested that changes in the overall cellular configuration of monomers, heterodimers, and homodimers could be altered by agonist as well as disease state, and this finding was supported by Lavoie et al. [47]. Also, *β*1-AR : *β*2-AR heterodimers have been shown to exhibit distinct functional and pharmacological properties, resulting in enhanced signaling efficiency in response to agonist stimulation and optimizing *β*-adrenergic modulation of contractility in cardiomyocytes [48]. However, controversy exists as to whether *β*-AR dimerization occurs in cardiovascular tissue. A report by Ianoul et al. [49] that used higher-fidelity imaging techniques suggested that *β*1-AR and *β*2-AR may be localized in two different populations of microdomains in cardiomyocytes, an observation inconsistent with the existence dimers. Of additional interest is a recent report by LaRocca et al. [50] that showed, also in cardiomyocytes, that *β*2-AR form heterodimers with the chemokine receptor type 4 (CXCR4) and this interaction negatively regulated isoproterenol-mediated *β*2-AR signaling by altering *β*2-AR sensitivity. Following from these studies, we evaluated whether CXCR4 activity modulation could alter *β*-AR-meditated vasorelaxation. Using aortae isolated from 2-month-old Fischer 344 rats, it appeared that CXCR4 activation inhibited, and CXCR4 blockade improved the vasorelaxant effect of isoproterenol (Figure 1). We also have found that Fischer 344 aortic vascular smooth muscle expresses CXCR4. Determination of age-related changes in CXCR4-mediated alterations in *β*-AR-stimulated vasoreactivity, CXCR4 expression, and the interaction between *β*2-AR and CXCR4 are underway.

### 3.2. G Proteins

The age-related change in *β*2-AR signaling appears to caused by changes in receptor sensitivity, and changes in G protein expression or function could manifest this physiology. Interestingly, as with *β*-AR expression, G*α*s expression similarly remains unchanged [52]. However, its function appears to be age impaired, as direct G*α*s activation-mediated cAMP production by was reduced in aortae isolated from old rats [53]. Our lab further characterized this observation by finding a marked decline in cholera toxin catalyzed ADP ribosylation labeling of G*α*s without a decline in the expression [54], suggesting some age-related alteration in G protein structure/function. We have also found that cholera toxin-mediated relaxation in aortic rings decreased with advancing age [7]. Further evidence that G*α*s function is altered with advancing age in the vasculature is from a study where it was attempted to reverse age-related declines in *β*-AR-mediated vasorelaxation by expressing a constitutively activated mutant of G*α*s (G*α*s-Q227L) into aorta from 6-month-old Fisher 344 rats. In that study [55], aorta that expressed G*α*s-Q227L exhibited enhanced isoproterenol-stimulated vasorelaxation, and both basal- and isoproterenol-stimulated cAMP production was increased. Therefore, G*α*s may undergo some age-related change that inhibits its ability to become activated via the action of an agonist-occupied receptor. In support of a functional change in G*α*s with age is the identification of a regulator of G protein signaling (RGS)/GTPase activating protein (GAP) that functions on G*α*s [26] called RGS-PX1. Age-related enhanced RGS-PX1 activity could impair *β*-AR signaling without changes in *β*-AR or G*α*s expression as agonist exposure would not initiate vasorelaxation as G*α*s signaling would be quenched due to the high RGS/GAP activity of RGS-PX1. At present, RGS-PX1 has not been evaluated within an aging paradigm. In total, the decline in *β*-AR-mediated vasorelaxation and cAMP accumulation observed in old vessels could be caused by a change in the function *but not* expression of G*α*s.

Another possible explanation for the decline in *β*-AR-mediated signaling could be an increase in G*α*i function, as this G protein subunit inhibits adenylyl cyclase activity and thus cAMP production. Also, the *β*2-AR, the predominant receptor species in vascular smooth muscle, has been shown in cardiomyocytes to rapidly link to G*α*i after agonist activation, and it is PKA-phosphorylated [38]. We actually found a slight decline in pertussis toxin labeling of G*α*i with age [54]. Also, a 30% decrease in G*α*i_1&2_ protein content between 6- and 24-month-old aortic preparations has been documented [52]. Age-related changes in G*βγ* could also affect *β*-AR-mediated signaling. G*βγ* has been shown to either stimulate or inhibit adenylyl cyclase activity in the presence of activated G*α*s [56]. G*βγ* also affects numerous plasma and organelle membrane-localized ion channels, thereby affecting the net polarity and potential for tonal changes of vascular smooth muscle [57]. However, we have found no age-related changes in the expression of G*βγ* subunit [54].

### 3.3. Adenylyl Cyclase, Protein Kinase A, cAMP, and Phosphodiesterases

As discussed, the fundamental change in blood vessels from older animals is a pronounced inability to relax to *β*-AR stimulation. This decline is directly correlated to an inability to synthesize appreciable concentrations of cAMP. However, old vessels do maintain the ability to relax entirely, as acetylcholine-, forskolin-, and nitrate-mediated vasorelaxation is complete [58]. Therefore, a probable protein candidate for the impairment is G*α*s (as discussed previous) or G protein receptor kinase (see following). The classical effector of G*α*s is adenylyl cyclase. Forskolin directly activates adenylyl cyclase, and thus stimulates cAMP production. Because forskolin stimulates blood vessels from young and old animals to relax completely, and to accumulate cAMP equally, it is generally thought that adenylyl cyclase function does not change with advancing age. Our results further support that adenylyl cyclase activity is maintained across aging [59]. However, the adenylyl cyclase family contains nine different isoforms, each with discrete tissue distribution [60]. Perhaps more importantly, each isoform is differentially regulated by various factors. For instance, calcium (at relevant intracellular concentrations) stimulates adenylyl cyclase subtype-1, and subtype-8 but inhibits subtype-3 and subtype-9. Interestingly, Zhang et al. have demonstrated that the predominant adenylyl cyclase isoforms in vascular smooth muscle are of the calcium-sensitive variety [61]. Therefore, a possible explanation for impaired receptor-mediated-cAMP production is not with the cyclase itself, but rather the interaction between cyclase and another, critical and age-affected, cellular factor that could regulate intracellular calcium sequestration.

Another line of reasoning would be that adenylyl cyclase activity is unchanged across age, but cAMP processing is altered. Therefore, changes in physiology could be due to age-related changes in the processing and degradation of cAMP through phosphodiesterases. We have determined that there are no age-related changes in general phosphodiesterase inhibitor-mediated vasorelaxation using 3-isobutyl-1-methylxanthine (IBMX), a nonspecific phosphodiesterase subtype inhibitory agent [62]. However, others [63] found that using a low dose of IBMX caused impaired cAMP accumulation in blood vessel preparation from older rats. The role of phosphodiesterases in mediating age-related changes in cAMP concentration is presently underevaluated. There are multiple phosphodiesterase subtypes, each that may have differential expression or activity [64]. Also, only recently have drugs specific to individual isoforms have become available. To that end, it has recently been shown that the vasodilator pathway associated with phosphodiesterase III is likely unchanged with aging in humans [65].

### 3.4. G Protein Receptor Kinases and Arrestins

*β*-AR desensitization is initiated by phosphorylation of the receptor, which is followed by its uncoupling from its signaling cascade. The kinases PKA, GRK, and others phosphorylate *β*-ARs [37]. Phosphorylated *β*-ARs are targets for another family of proteins that mediate uncoupling/desensitization, the arrestins [24]. Therefore, arrestins function in concert with GRKs to attenuate intracellular signaling [66, 67]. To date, six different GRKs have been identified. Of interest, GRK-2, GRK-3, and GRK-5 (GRK-2 and GRK-3 are also known as *β*-AR kinases: *β*-ARK-1 and *β*-ARK-2, resp.) target *β*-ARs and are highly expressed in the cardiovascular system [23].

Evidence shows that GRKs are important regulators of pathology in humans [68]. Significant increases in GRK activity and expression have been observed in ventricles of failing human hearts [69]. The progression of Alzheimer's disease has also been associated with enhanced GRK function in fibroblasts taken from human skin [70]. Relating specifically to GRK function and the vasculature, cultured vascular smooth muscle cells have been shown to express GRK-2 both within the cytoplasm and at the membrane [71], and a transgenic mouse that overexpressed GRK-2 in a vascular-specific manner has been developed [72]. These mice exhibited attenuated *β*-AR-mediated cAMP production and vasorelaxation. Also, overexpression caused elevations in resting blood pressure and was accompanied by an increase in vascular thickening, suggesting a decline in cAMP generation.

An age-related change in GRK activity or expression in vascular tissue would implicate GRKs in the age-related decline in *β*-AR mediated vasorelaxation. Only a few studies have been performed to assess age-related changes in GRKs. No changes in GRK activity or GRK-2 and 5 expression were observed in lymphocytes of aged humans [73]. However, expression of soluble GRK-2 increased with maturation in thoracic aortic preparations from Fischer 344/Brown Norway rats [11]. We have also examined age-related changes in GRKs [51]. In aorta from aged Fischer 344 rats, total GRK activity increased nearly 2.1-fold. In the soluble (cytosolic) fraction, GRK-2 expression increased nearly 3.6-fold, GRK-3 expression increased approximately 3.8-fold, and *β*-arrestin expression increased approximately 1.6-fold. In the membrane fraction, GRK-2 expression increased approximately 1.5-fold, GRK-3 expression increased nearly 2.1-fold, while there was not an age-related change in the expression of GRK-5. These data suggest that a critical feature of age-related impaired *β*-AR signaling may be imparted through an increase in total pool of GRK that could be explained by either increased expression, or decreased degradation [74]. This increased pool could allow for enhanced targeting of these receptor kinases to the membrane, and hence the *β*-AR. Whether *β*-ARs from aged vessels have increased phosphorylated residues, and thus enhanced desensitization is yet to be established. Also, the mechanism for the enhanced expression of GRKs with advancing age is likewise yet to be explained but is an active interest of our lab.

### 3.5. Scaffolding

*β*-AR signaling depends on the interaction between numerous proteins. Therefore, *β*-AR-mediated function requires appropriate localization (cytoplasmic versus membrane) and organization (to allow efficient and rapid interaction with one another) of these proteins. Therefore, recent research has focused on “scaffolding proteins,” which are intracellular proteins that compartmentalize multiple related signaling molecules to specific intracellular domains. This theory has replaced the classical “random collision-coupling theory” of signal transduction [75]. The *β*-AR signaling cascade is anchored within the plasma membrane by the scaffolding protein caveolin [76]. *β*_2_-ARs [77, 78], numerous G-proteins (including G*α*s) [79, 80], adenylyl cyclase (numerous isoforms) [80–82], and GRKs 2, 3, and 5 [83] all localize in caveolin-rich domains of the cell membrane. 

Age-related changes in caveolin have recently been demonstrated revealing tissue-specific changes in expression [84]. Also, our results with Fischer 344 rat aortic tissue show that the expression of caveolin-1 decreases with advancing age [85]. An age-related change in caveolin expression could easily alter the milieu of proteins within a *β*-AR signaling pocket, and thereby alter signaling. Indeed, Carman et al. [83] found that GRK activity is inhibited when GRK is bound to caveolin-1. Our lab has shown that with advancing age, the interaction between caveolin-1 and GRK2 substantially declines [85]. Therefore, both the scaffolding and protein activity-modulating functions of caveolin may be compromised with advancing age in the vasculature. 

Other reports implicate caveolin as a regulator of vascular function [86]. Razani and Lisanti [87], and Drab et al. [88] produced caveolin-1 null mice and found that these animals exhibited impaired aortic steady-state maximal tension induced by phenylephrine (an *α*-AR agonist). Also, acetylcholine-mediated (nitric oxide-dependent) vasorelaxation was similarly altered in null animals. Finally, caveolin-1 null mice displayed hyperproliferation in certain cell types, suggesting (but not documented) a decline in cAMP production. Changes in *β*-AR-, cAMP-, or age-mediated effects in the caveolin-1-null mice have yet to be evaluated. However, these data from transgenic animals clearly indicate that caveolin is an important modulator of vascular function.

### 3.6. Receptor Cross-Talk and Ion Channels

A potentially interesting phenomenon observed in molecular signaling is receptor cross-talk events. Cross-talk between G*α*q-linked receptors and *β*-ARs have been observed. Activation of PKC by G*α*q-linked-agonists directed GRKs to the membrane, enhancing *β*-AR phosphorylation and desensitization [89]. Also, it has been shown that GRK-2 is more effective at desensitizing *β*-ARs after its activation by PKC [90]. Finally, antisense technologies have been used to knockout PKC expression and function; these studies determined that this manipulation produced enhanced *β*-AR agonist-induced desensitization rather than the expected attenuation result. In addition, these authors subsequently found that this effect was linked to phosphatase activity [91]. These findings suggest that PKC might also be involved with *β*-AR resensitization through interaction with a phosphatase. Therefore, phosphorylation/dephosphorylation and desensitization/resensitization of *β*-ARs can be induced from a number of stimuli including angiotensin II.

In terms of vascular *β*-AR being altered by receptor cross-talk are *in vitro* studies showing that angiotensin II enhanced *β*-AR-mediated cAMP production in cultured aortic vascular smooth muscle cells [61, 92, 93] as well as in preglomerular microvascular smooth muscle cells [94, 95]. In terms of vasorelaxation being affected was a study that found that angiotensin II can enhance cAMP-mediated vasorelaxation via angiotensin II-type 1-receptors (AT_1_) [96]. We examined the interaction among aging, *β*-AR-mediated vasorelaxation, and angiotensin II [62]. Our results showed that this effect of angiotensin II on agonist-mediated vasorelaxation was limited to young (6-week-old) or adult (6-month-old) rats, was absent in aged (12- and 24-month-old) animals, and was mediated by angiotensin II-type 1 receptors. Angiotensin II appeared to amplify vasorelaxation in aorta from 6-week and 6-month-old animals via enhanced production of cAMP. The mechanisms involved with angiotensin II enhanced, *β*-AR-mediated signaling are unknown but may involve adenylyl cyclase, G*α*s, or calcineurin. Further study may show that aging may effect a factor common to both angiotensin II and *β*-AR signaling pathways or that aging may impair cross-talk between these two receptor pathways. 

A final interesting aspect of age-related changes in *β*-AR-mediated signaling is understanding the role of various ion channels; it is well understood that the function of numerous ion channels is responsible for determining membrane potential [97]. The effect of isoproterenol on the ionic milieu of aortic vascular smooth muscle cells was characterized [98]. Results determined that isoproterenol functioned by inducing hyperpolarization via activating ATP-sensitive potassium channels (K_ATP_). They also determined that the isoproterenol/K_ATP_-mediated hyperpolarization was impaired in smooth muscle cells from older rats. However, the effect of direct activation of K_ATP_ was unchanged between young and old groups. Therefore, their data fit well with what has been previously known about the age-related changes in *β*-AR signaling—the alteration appears to be localized proximal to adenylyl cyclase and may involve changes in the *β*-AR itself or in its ability to couple to other regulatory molecules.

### 3.7. Endothelium-Localized *β*-AR/VSM Interactions

A controversial topic is the endothelium-mediated effect on the age-related change in *β*-AR function. It is clear that age-related changes in the endothelium occur [99], and there are endothelium-localized *β*-AR [100]. It is also well accepted that removal of endothelium reduces the effect of isoproterenol on vasorelaxation in a variety of isolated arteries and veins from different species, including humans [100]. Data show that endothelial cells have binding sites for *β*-AR ligands [101] and that isoproterenol increases nitric oxide synthase activity in these cells. Compatible with these findings is that inhibition of nitric oxide synthase modestly decreased relaxation to *β*-AR agonists [102]. Therefore, endothelium- and vascular smooth muscle-mediated function may be additive in that *β*-AR-mediated vasorelaxation appears to be induced via both nitric oxide-mediated pathways (endothelial), and cAMP-mediated pathways (vascular smooth muscle) [103].

In terms of aging, *β*-AR vasorelaxation is initiated in both endothelial and vascular smooth muscle cells. However, [104] found that the endothelial component did not change with age, whereas the vascular smooth muscle component did. They did identify an age-specific, endothelium-dependent effect in that vascular tone appeared to be mediated through an endothelium-derived hyperpolarizing factor (tetraethylammonium-sensitive K+ channels) that was increased with advancing age. Age-related changes in membrane polarization are also discussed previously where K_ATP_-mediated hyperpolarization was found to be impaired in vascular smooth muscle cells from older rats [98]. Our lab has also produced data in support of a role for changes in polarization in mediating the age-related change in *β*-AR-mediated vasorelaxation. When comparing the effect of age on isoproterenol-mediated vasorelaxation on phenylephrine- versus KCl-contracted aorta, vessels contracted with phenylephrine relaxed to a substantially higher degree that those contracted with KCl, although the age-effect was maintained [7]. One interpretation of this result is that with advancing age, isoproterenol-mediated signaling pathways involve an increased role for K+ channels. Further support that the aging change in *β*-AR signaling is vascular smooth muscle-, rather than endothelium-dependent is that other agents that initiate vasorelaxation through vascular smooth muscle localized G protein-coupled receptors (adenosine, parathyroid hormone) also show impaired vasorelaxation with age [105, 106]. Therefore, the age-related change is likely due to a factor common to all vascular smooth muscle-localized G protein-coupled receptors, such as GRK (see above discussion), while a nonage-related endothelial dependent component contributes to *β*-AR-stimulated vasorelaxation in general.

## 4. Summary and Perspectives

Hypertension, orthostatic hypotension, arterial insufficiency, atherosclerosis, and restenosis are common disorders in the elderly that lead to significant morbidity and mortality. These clinically significant conditions all may have a common feature in that they are associated with and age-related change in *β*-AR signaling. Impaired *β*-AR-mediated vasorelaxation with age is observed throughout species and arterial beds, and in aged vascular tissue, *β*-ARs are desensitized. One cellular process that changes with age that modulates *β*-AR sensitivity is phosphorylation by GRK-2. But, there are multiple other protein factors that may modulate *β*-AR function with aging. Similarly, there are multiple factors that may modulate GRK function [107]. Therefore, the overall complexity of the molecular pathways creates difficulties in isolating a single therapeutic target. Regardless, a large and growing segment of the general population are age 65 or older, and this percentage will continue to rise. Optimal care of this population is a priority for clinicians, and better understanding of this age-related change in the vasculature will allow for innovative strategies for the management of multiple disorders. Findings will be applicable to other tissues and disease states where *β*-AR signaling is altered (such as in the kidney and liver [108], heart [109], lung [110], and brain [111].
